# Adequate Utilization of Emergency Services in Germany: Is There a Differential by Migration Background?

**DOI:** 10.3389/fpubh.2020.613250

**Published:** 2021-01-08

**Authors:** Odile Sauzet, Matthias David, Baharan Naghavi, Theda Borde, Jalid Sehouli, Oliver Razum

**Affiliations:** ^1^Department of Epidemiology and International Public Health, Bielefeld School of Public Health, Bielefeld University, Bielefeld, Germany; ^2^Centre for Statistics, Bielefeld University, Bielefeld, Germany; ^3^Department of Gynecology With Center for Oncological Surgery, Virchow Campus, Charité University Hospital, Berlin, Germany; ^4^Charité Comprehensive Cancer Center, Campus Charité Mitte, Charité University Hospital, Berlin, Germany; ^5^Alice Salomon University of Applied Sciences, Berlin, Germany

**Keywords:** migration, access to services, emergency services, low urgency, adequacy of use

## Abstract

**Background:** The role of emergency services (ES) is to provide round-the-clock acute care. In recent years, inadequate use of ES has been internationally thematised because of overcrowding and the associated cost. Evidence shows that migrant populations tend to use more ES than non-migrant but it remains to show if there is a differential in inadequacy.

**Method:** Quantitative data from consecutive patients visiting three ES in Berlin (hospital-based outpatient clinics for internal medicine or gynecology) from July 2017 to July 2018 were obtained. Utilization was defined as adequate if the patient was admitted to hospital and/or if all of the three following criteria were fulfilled: reported to have been sent by medical staff; reported strong pain; and reported a high urgency (both ≥7, scale from 0 to 10). Differences between migrants (1st generation), their offspring (2nd generation), and non-migrants were evaluated using logistic regression.

**Results:** Of the 2,327 patients included, 901 had a migration background. Adjusting for gender, age, gynecological hospital-based outpatient clinic, and the number of chronic diseases, 1st generation migrant patients (*n* = 633) had significantly lower odds than non-migrants to have an adequate utilization of services [OR 0.78, 95% confidence interval (0.62, 0.99), *p*-value 0.046]. For 2nd generation patients (*n* = 268), no statistically significant difference was found [OR 0.80, 95% confidence interval (0.56, 1.15), *p*-value 0.231]. Only adjusting for gynecological hospital-based outpatient clinic did weaken the association between migration status on adequacy but interactions between type of hospital-based outpatient clinic and migration were not significant.

**Discussion:** First generation migrants show lower odds of adequate ES use compared to non-migrants. Only visiting a gynecological hospital-based outpatient clinic as opposed to internal medicine could partly explain the lower odds of adequate use among immigrants. This indicates a need for structural changes in the healthcare system: The threshold of access to general practices needs to be lowered, considering the needs of diverse subgroups of migrant patients.

## Background

A medical urgency can be defined as a change in the physical or mental condition of a person for which an immediate medical attention is deemed necessary by the patient or a third person (1). The role of an emergency service (ES) is to provide, right away, seriously ill patients or patients classified as an urgent case with the appropriate medical attention by specially trained staff using the resources of a hospital. The characteristic of ES is the round the clock availability with low threshold of accessibility. For example, German ES pose almost no restriction to access compared to doctor practices which are open only during day time and (in most cases) require appointments. Internationally, overcrowding and overuse of ES threaten the quality of care and the safety of patients (2).

Adequacy of use of emergency services is not well defined, and a range of criteria have been used in the literature. These criteria involve an expert opinion, hospitalization, or less frequently the patient's own estimation of urgency (3). A review of the literature comparing emergency service use between migrant and non-migrant populations in Europe reported that the main criterion used to assess the adequacy of use was either triage categories or the costs incurred by treating the patients (4).

In Germany, ES treat around 20 million patients per year (5) but only some 40% of them are subsequently admitted to hospital (6). The number of visits to ES in German hospitals has been increasing constantly in the last years in particular by patients with low urgency (7, 8). According to the estimation of one of the statutory health insurances (9), about a third of emergency patients could be treated by a general practitioner. The speed at which a diagnostic is obtained and high quality of care in ES as well as the ease of access may explain why patients in non-urgent cases use these services (3, 10). In a survey in Berlin in 2010, 57% of the patients reported having tried to get in touch with a medical doctor in a practice during opening hours (11).

Because the use of medical services is related to both accessibility and need, which may vary between patients from different socio-economic groups or migration background, differences in pattern of use may occur with causes related to health status, perceived needs, language barrier, cultural differences, or (migration related) trauma (12). Higher use of ES among migrants compared to non-migrants can be observed across Europe (13). The review by Norredam et al. (12) showed a slightly higher use of ES by persons with migration background but did not address the question of a differential in adequacy of use. A study in Italy of children under the age of one showed that differences in frequency of use between migrants and non-migrants were present only for low urgency indicating a lower adequacy of use by migrants (14). In Germany a descriptive cross-sectional study in the years 2001–2002 has shown evidence of an increased inadequate utilization of ES by migrants compared to non-migrant (15) and hypothesized that it could be explained through cultural and language differences rather than by a differential in medical needs.

Adequacy of use of emergency services is not well defined, and a range of criteria have been used in the literature. These criteria involve an expert opinion, hospitalization, or less frequently the patient's own estimation of urgency (8). A review of the literature comparing emergency service use between migrant and non-migrant populations in Europe reported that the main criterion used to assess the adequacy of use was either triage categories or the costs incurred by treating the patients (15).

In this work, we first define a criterion for adequacy based on an expert opinion approach. Then we find out if in a context of a general increase in use, a differential in adequate utilization of ES for patients in the lower triage categories exits between migrant and non-migrant patients in the city of Berlin, Germany and evaluate the role of potential explanatory factors to obtain new hypotheses.

## Method

### Data

Consecutive patients visiting three ES of large hospitals in Berlin/Germany (Charité, Campus Virchow-Klinikum Charité (Department of Gynaecology and Centre of Gynaecological Oncology only); Campus Benjamin Franklin; Vivantes Klinikum Neukölln, both ES specialized in internal medicine) from July 2017 to July 2018, were recruited into the study by a trained study nurse or interviewer who led the interviews. In Germany emergency services are provided not by GPs, but by specialist doctors in hospital-based outpatient clinics. A written consent was obtained for all patients involved in the study. The interviewers were not involved in the medical management of the patients. Inclusion criteria were every patient who were conscious and in no life-threatening condition. Exclusion criteria were being under the influence of alcohol or not being responsive, being under 18 year of age or not being able to answer the questionnaire in any of the languages available (German, Turkish, Arabic, and English).

A structured questionnaire developed for a previous study (16) has been adapted and tested. It contains questions about the reasons for attending ES, previous visits, socio-economic as well as migration related questions. Information about the country of birth of the participants and their parents was collected to assess the migration status of patients according to Schenk et al. (17). Moreover, routine hospital data concerning the diagnostic and therapy plan of patient was obtained. The physicians who treated the participating patients were provided with a questionnaire in which an estimation of the urgency of the medical condition was given.

The study was reviewed and approved by the ethics commission of the Charité (EA 2/102/17 Ethikkommission der Charité, Ethikausschuss am Campus Virchow-Klinikum).

### Establishing Criterion to Determine Adequacy of Use

Due to a limited return of physician questionnaires and the lack of an objective or recognized criterion, we developed a model to predict physician assessment of urgency based on questions available on the patient questionnaire (estimation from the physician of an urgency of seven or higher on a scale from 0 to 10) and/or hospital admission. This meant finding which combination of variables from the patient questionnaire (available for all cases) could best predict adequacy (defined as either admitted to hospital and/or with high estimation of urgency by the physician).

### Statistical Analysis

The aim was to establish if there were associations between adequate use of ES (binary outcome: criterion satisfied yes/no) and migration status (migrant 1st generation – born abroad –, 2nd generation – at least one parent born abroad –, non-migrant) and find out which factors could affect possible associations. For this purpose, we fitted logistic regression models. The first model adjusted only for confounding variables gender, age (continuous) and the number of chronic diseases (none, 1, 2, 3 or more), then variables were added in increment to subsequent models: type of facility (gynecology/internal medicine, because of differences in accessibility compared to general practice) was added in Model 2. In Model 3 we added variables relative to the motivation for using a specific hospital: which medical condition motivated the hospital visit as stated in the hospital records (13 categories, listed in the Supplementary Material), the reason for choosing the hospital visited, and if the visit occurred after 19:00 and before 8:00. And finally Model 4 added socio-economic factors (employment status and educational attainment). Interactions between all explanatory variables (except with the type of medical condition) and migration status were also tested if possible due to sample size.

Education achievement was collected using six categories which we categorized in three: low (primary education), middle (vocational qualification), high (high school degree and above). Employment status was recorded as a binary outcome. Patients were provided with a list of potential chronic diseases plus free text from which we calculated the number of chronic diseases. The medical conditions were obtained from medical records as documented by the physician during the visit and concern the present condition, which motivated the hospital visit. These conditions were categorized by medics working on the project. Possible reasons to choose the hospital were: recommendation from medical staff (including referral but not just), being taken there by ambulance, after recommendation from friend/family, due to its reputation, known as the relevant facility for condition, or because it was nearby. We re-categorized these to proximity of location, sent by medical staff, reputation/recommendation, known or relevant facility, and other.

Type of facilities, medical condition and time of the visit were the only variables used in the regression models, which are not patient reported but reported from hospital records or the interviewer.

## Results

### Population

**Figure 1:**
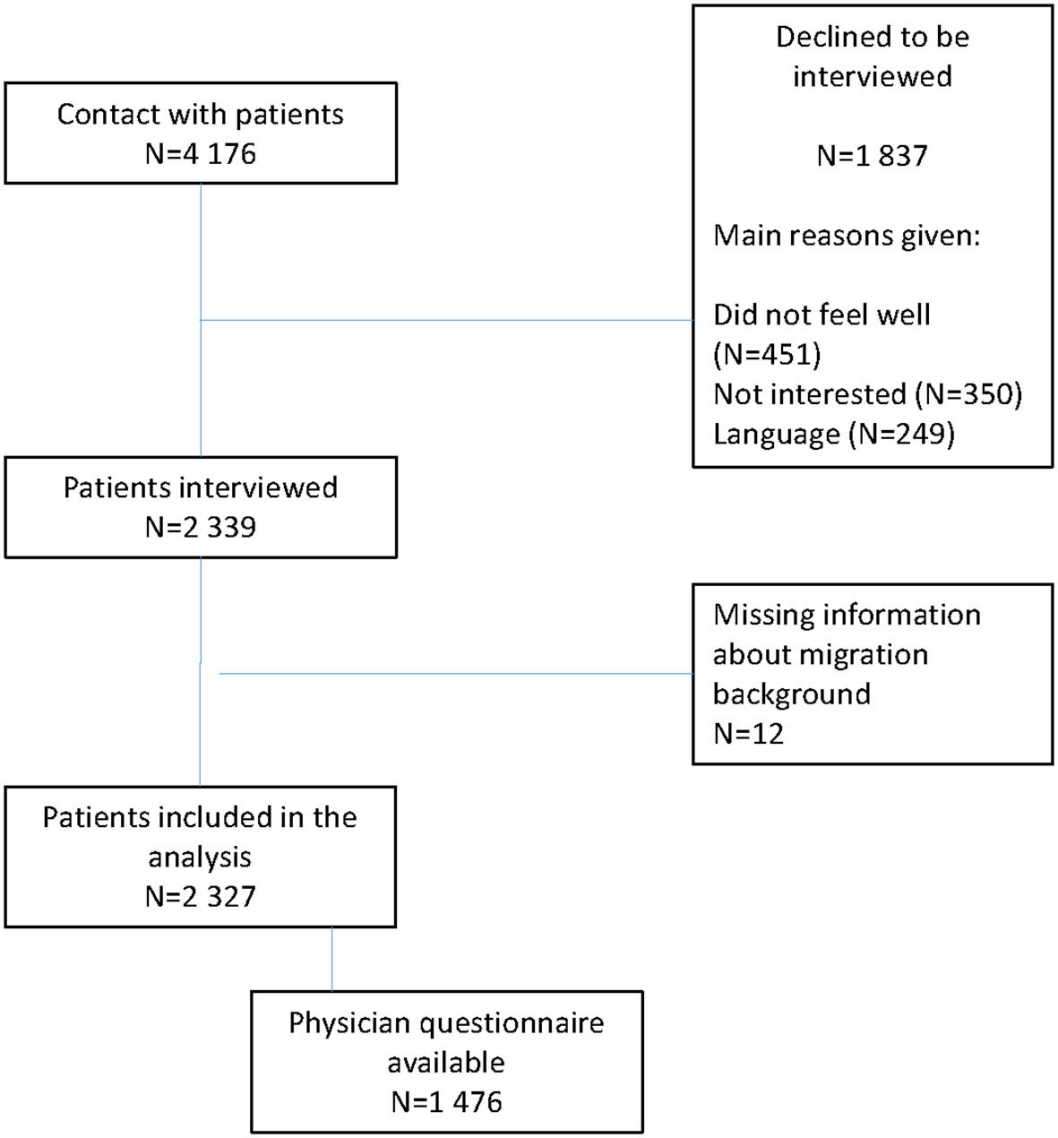
Flow chart.

A total of 4,176 Patients were approached of which 2,339 (56%) agreed to participate. The most common reasons given not to participate were being too unwell (*n* = 451), no motivation (*n* = 350) and language difficulties (*n* = 249). The average duration of interviews was 20 ± 8 min. A flow chart is given in Figure 1. The majority of the patients were recruited from internal medicine hospital-based outpatient clinics (*n* = 1,596), while the others (*n* = 731) visited a specialist gynecological hospital-based outpatient clinic.

The migration status could be established for 2,327 participants, of which 61% (1,426) did not have a migration background, 633 (27%) were classified as migrant 1st generation (born in a foreign country) and 268 (12%) as 2nd generation migrant (born in Germany from at least one parent born in a foreign country). This distribution reflects the data from the 2019 micro-census for Berlin, with a small over representation of people with migration experience: 66% of the Berlin population had no migration background and 22% had a migration experience (18).

The most common area of origin for the 1st generation migrants was the European Union with 34% (216/626) of respondents followed by Turkey (15%, 96/626) and Russia, non-EU eastern Europe, or former Soviet Union (15%, 94/626). Other regions included the Middle East, North and South America, Asia and Africa. This was estimated by the nationality provided or the country of birth for those with German nationality.

Descriptive statistics of the participants' profile is given in Table 1. Non-migrant participants were on average older (60 years standard deviation 21) than migrants [1st generation: 42 years (16), 2nd generation 32 (12)]. There was a large majority (70%) of female participants (explained by one ES being specialized in gynecology).

**Table 1:** Descriptive statistics with mean (standard deviation) for continuous variables and frequencies (percentage from total in the subgroups).

	**Non-migrants (*N* =1,426)**	**Migrants 1st generation (*N* = 633)**	**2nd generation (*N* = 268)**	**Total (*N* = 2,327)**
Age (years)	60 (21)	42 (16)	32 (12)	52 (22)
Gender (female)	886/1,424 (62%)	488/633 (77%)	226/267 (85%)	1,600/2,325 (69%)
Type of emergency out-patient clinic				
Gynaecology^**^	248/1,426 (17%)	333/633 (53%)	150/268 (56%)	731/2,327 (31%)
Reason for choosing this particular services (patient reported)				
Decided by a health professional	488/1,426 (34%)	140/633 (22%)	45/268 (17%)	673/2,327 (29%)
It was geographically close	473/1,426 (33%)	261/633 (41%)	109/268 (41%)	843/2,327 (36%)
Reputation/recommendation from friend or family	51/1,426 (4%)	45/633 (7%)	16/268 (6%)	112/2,327 (5%)
Have been there before/competent for my condition	382/1,426 (27%)	166/633 (26 %)	93/268 (35%)	641/2,327 (28%)
Other	32/1,426 (2%)	21/633 (3%)	5/268 (2%)	58/2,327 (2%)
Evening/night	115/1,426 (8%)	51/633 (8%)	24/268 (9%)	190/2,327 (8%)
Education Reason for choosing this particular services (patient reported)				
High	376/1,419 (27%)	288/625 (46%)	86/268 (32%)	750/2,312 (32%)
Medium	778/1,419 (55%)	202/625 (32%)	136/268 (51%)	1,116/2,312 (48%)
Low	2,651,419 (19%)	135/625 (22%)	46/268 (17%)	446/2,312 (19%)
Employed (patient reported)	502/1,425 (35%)	283/631 (45%)	154/268 (57%)	939/2,324 (40%)
Chronic diseases (patient reported)	1,072/1,426 (75%)	303/633 (48%)	122(268 (46%)	1,497/2,327 (64%)
Visit of emergency services in the last year (patient reported)				
Once	350/1,411 (25%)	146/627 (23%)	69/264 (26%)	565/2,302 (25%)
Twice	127/1,411 (9%)	70/627 (11%)	35/264 (13%)	232/2,302 (10%)
Three times or more	159/1,411 (11%)	56/633 (9%)	35/264 (13%)	250/2,302 (11%)
a. Admission to hospital^**^	656/1,365 (48%)	157/584 (27%)	49/257 (19%)	862/2,206 (39%)
b. Pain (≥7 on a 0–10 scale)	326/1,426 (23%)	229/633 (36%)	95/268(35%)	650/2,327 (28%)
c. Patient reported urgency (≥7 on a 0–10 scale)	758/1,402 (54%)	408/606 (67%)	164/266 (61%)	1,330/2,274 (58%)
d. Advised by medical staff	585/1,418 (41%)	198/631 (31%)	84/268 (31%)	867/2,317 (37%)
b. c. d. satisfied	94/1,422 (7%)	51/629 (8%)	18/268 (7%)	163/2,319 (7%)
Adequacy of utilization of services^*^	675/1,426 (47%)	161/633 (25%)	51/263 (19%)	887/2,327 (38%)

* *Criterion used in the analysis: a. and/or patient reported (b., c., and d.) are satisfied*.

** *Physician reported or from interview*.

#### Patient Based Criterion to Determine Adequacy of Use

From the 1,476 returned physician questionnaires, a total of 1,392 presented an estimation of urgency. Urgency was estimated as high by the physician in 22% of cases, with substantial disparity between migration statuses: 28% of those with no-migration background (from 849), 14% of those with migration experience (from 391) and 11% of the second generation (from 152) urgency was estimated as high. The best combination of variables to predict physician high urgency and/or admission to hospital required all of the three following to hold: a. the patient reported a degree of pain of 7 or more (on a scale from 0 to 10), b. the patient reported that they were advised by medical staff to visit the ES, and c. the patient reported an urgency degree of 7 or more (on a scale from 0 to 10). The proportion of false positives was 1.4% (criterion fulfilled, but no admission or judgment of urgency from the physician) and of false negatives 8.8% (admission to hospital and/or judgment of urgency from the physician but criterion not fulfilled).

The final adequacy criterion which was available to all patients in the study is: admission to hospital and/or if all of the three following hold: a. the patient reported a degree of pain of 7 or more (on a scale from 0 to 10), b. the patient reported that they were advised by medical staff to visit the ES, and c. the patient reported an urgency degree of 7 or more (on a scale from 0 to 10). The proportion of patients fulfilling adequacy criterion are provided in Table 1. Admission to hospital dominates the other criterion as only 25 patients are classified as having an adequate use but were not admitted to hospital.

#### Regression Analyses

For all four models there was a lower odds of an adequate use of services for both 1st and 2nd generation migrants but it reached significance only for the 1st generation (Table 2). Increasing age was a major predictor of adequacy of utilization and visiting a gynecological hospital-based outpatient clinic compared to a clinic for internal medicine a major predictor of inadequacy (see Supplementary Table).

**Table 2:** Results of the regression analysis for migration status.

**Adequate use of services**	**Model 1 (*****N*** **=** **2,287)**			**Model 2 (*****N*** **=** **2,287)**			**Model 3 (*****N*** **=** **2,287)**			**Model 4 (*****N*** **=** **2,271)**		
	**OR**	95% CI^**^	***p*-value**	**OR**	95% CI^**^	***p*-value**	**OR**	95% CI^**^	***p*-value**	**OR**	95% CI^**^	***p*-value**
**Migration (reference: non-migrant)**												
1st generation	0.72	(0.57, 0.91)	0.005	0.78	(0.62, 0.99)	0.046	0.78	(0.61, 0.99)	0.047	0.77	(0.60, 0.99)	0.046
2nd generation	0.76	(0.53, 1.09)	0.131	0.80	(0.56, 1.15)	0.231	0.76	(0.50, 1.05)	0.093	0.72	(0.50, 1.03)	0.077

*All variables are patient reported to the exception of type of facility and Evening/night, which was provided by the interviewer. All models are adjusted for gender, age (continuous) and the number of chronic diseases. Model 2 added the type of facility (gynecology/internal medicine) to model 1. Model 3 added the reason for choice of facility, if day or night, and the type of condition that motivated the visit to the emergency services (13 categories) to model 2. Model 4 added education level and employment status to model 3*.

** *Confidence interval*.

In Model 1 which adjusted only for gender, age and the number of chronic diseases, the odds of adequate use by 1st generation migrants relative to non-migrants were 0.72 [95% confidence interval: (0.57, 0.91), *p*-value: 0.005] and for 2nd generation 0.76 [95% confidence interval: )0.53, 1.09), *p*-value: 0.131].

Adjusting for gynecological hospital-based outpatient clinic as opposed to internal medicine (Model 2) attenuated the association of migration status on adequacy: 0.78 [95% confidence interval: (0.62, 0.99), *p*-value: 0.046] and for 2nd generation 0.80 [95% confidence interval: (0.56, 1.15), *p*-value: 0.231].

In Model 3 adding conditions which led to attend the ES and the motivation for choosing this particular ES only changed marginally the odds of adequacy (see Table 2).

In Model 4 which additionally adjusted for socio-economic factors, including educational attainment and employment status also changed the odds of adequacy only marginally compared to Model 2 (see Table 2). None of the interactions tested were significant which could be due to a lack of power.

A sensitivity analysis showed that a reduction in the number of criteria to fulfill the adequacy criterion from three to two did not change the results of the comparison between migration statuses. Estimates for covariates are provided in the Supplementary Table.

## Discussion

The aim of this study was to find out whether there was a differential in appropriate use of ES between migration groups (no-migration, 1st generation, 2nd generation) with data from three ES in Berlin, Germany as well as which factors influenced these associations. After controlling for possible explanatory variables and confounder including health needs, a significant lower odds of adequate utilization of services remained for migrants 1st generation (not born in Germany) compared to non-migrants. For 2nd generation persons (at least one parent not born in Germany) a similar association did not reach significance maybe due to a lack of power. While employment, education, and geographical proximity were also predictors of adequacy, no interaction with migration status could be seen and adding these variables to the models did not change the association with migration status. Only visiting a gynecology emergency clinic compared to internal medicine did modify the size of the association between migration status and adequacy. More detailed results for patients in the present study who visited the gynecology hospital-based outpatient clinic are reported in (19).

The observed rate of admission to hospital (40%) was in line with what has been reported elsewhere (6). Due to methodological constraints, however, comparability is limited. This study confirms the findings of David conducted in 2006 (15) concerning a decreased rate of adequacy among 1st generation migrants. The differentials in adequacy could not be explained by either the medical condition of the patients, nor their socio-economic status. However, our findings indicates that the accessibility of some specialist practices like gynecology may play a role.

The common hypothesis that inadequate use of ES is linked to a lack of availability of out-of-hours general practices could not be confirmed with this study because most of the participants included visited the services during day-time. Moreover, we were not able to see an association with day/evening-night ES visits on adequacy of utilization.

A possible explanation for the differences observed is in how pain and urgency are perceived by patients with a migration experience. As seen in Table 1, patients with a migration background have a higher rate of reported high pain or perceived urgency for medical treatment than patients without migration background. However, there is a lower rate of patients reporting having been recommended to visit ES by medical staff among patients with migration background as for those without. Therefore, more visits by patients with migration background were classified as inadequate. These differences in perception cannot just be explained through cultural differences because of the heterogeneity of origin of the patients collective but may be related to having experienced migration. As shown by the role of gynecology hospital-based outpatient clinic in this work, we hypothesize that the association seen could be related to the real and perceived accessibility of medical practices for persons with a migration experience as opposed to the low accessibility barrier of ES. However, this must be further investigated.

A limitation of our study is that differences due to health literacy or knowledge of the health system could not be assessed. Another limitation is the way adequacy of use was defined; there is not yet an agreed definition of this concept. But a review of studies of adequacy of use of ES (3) showed that admission to hospital was the most commonly used criterion. German knowledge was not a significant determinant of adequacy of use in our study when adjusted for migration status. But language barriers being a reason for non-participation we cannot make any conclusive remark about any association with language knowledge.

Other limitations include the high proportion of refusal to participate for which we cannot assess if the reasons given were associated with migration status. The reason is that the migration status from those who declined to participate is not known. It could be a source of bias if patients with migration experience were more likely to refuse to participate because they were more severely ill than other groups. This could bias the results toward more inadequacy. However, the distribution of migration status in our sample reflects the one for Berlin as a whole, making this bias unlikely. We also were unable to properly test for interaction between factors affecting the adequacy of use and migration status due to small sample sizes. For that matter, we could only assess if controlling for these variables did change the relationship between adequacy and migration status. We presented odds ratios, which cannot be interpreted as risk ratios because of the outcome is not rare and this study does not provide estimates of incidence of inadequacy. However, the aim of the study was to assess the existence of associations and factors, which might influence these associations. This can be done with logistic regression models. Because of limited sample size, we could not assess any association with the region of origin.

The definition of ES as the provision of care for seriously ill or injured persons does not reflect the use patients make of the services. Our study shows that patients with migration experience tend to show less adequate use of ES for reasons other than differences in socio-economic background or health needs.

Despite efforts to provide information about the health care system in a variety of languages, our analyses shows that there is no evidence that the health care system has adapted to the diversity of its service users leading to a part of the population to either over-utilize easier-to-access services or not being offered the services which would correspond to their needs. This is confirmed by the fact that some part of the decreased odds of adequacy for migrant 1st generation is explained by the choice of specialist gynecology hospital-based outpatient clinic as opposed to internal medicine indicating difficulties to access gynecological practices by these populations. Possible action would be to provide low barrier hospital based practices for which no appointment would be necessary as it is already done in some cities across Germany.

## Conclusion

There is a discrepancy between the current purpose of ES and the needs of a diverse patient population with varying expectations, language proficiencies, health literacy and health conceptions. New forms of inclusive health care provisions with low accessibility thresholds as well as new information strategies by the statutory health insurance organizations informing their members about the health care system should be considered to reduce the inadequacy of use of ES and improve the accessibility of health care provision for a diverse population.

## Data Availability Statement

The datasets presented in this article are not readily available because of confidenciality reasons. Requests to access the datasets should be directed to the authors.

## Ethics Statement

The studies involving human participants were reviewed and approved by Ethics commission of the Charité (EA 2/102/17 Ethikkommission der Charité, Ethikausschuss am Campus Virchow-Klinikum). The patients/participants provided their written informed consent to participate in this study.

## Author Contributions

MD, OR, and JS designed the study. OS and BN collected and analyzed the data. OS drafted the manuscript with TB and MD. All authors contributed to the article and approved the submitted version.

## Conflict of Interest

The authors declare that the research was conducted in the absence of any commercial or financial relationships that could be construed as a potential conflict of interest.

## Abbreviations

ES: Emergency services.
