# Supplementary material for: Adequate Utilization of Emergency Services in Germany: Is There a Differential by Migration Background?
Source: Front Public Health. 2021 Jan 8;8:613250. doi: 10.3389/fpubh.2020.613250 (PMC7820806; doi:10.3389/fpubh.2020.613250)
Supplement: Supplementary file 1 [file Data_Sheet_1.PDF]

	Migration Status (n, %)			
Condition	None	1st generation	2nd generation	Total
Internal pain	**310**<br>**21.74**	**174**<br>**27.49**	**76**<br>**28.36**	**560**<br>**24.07**
Back pain	**59**<br>**4.14**	**18**<br>**2.84**	**7**<br>**2.61**	**84**<br>**3.61**
Gynecological problems	**122**<br>**8.56**	**167**<br>**26.38**	**86**<br>**32.09**	**375**<br>**16.12**
Dermatological symptoms	**72**<br>**5.05**	**33**<br>**5.21**	**13**<br>**4.85**	**118**<br>**5.07**
Urological Symptoms	**18**<br>**1.26**	**8**<br>**1.26**	**6**<br>**2.24**	**32**<br>**1.38**
Respiratory problems	**289**<br>**20.27**	**79**<br>**12.48**	**24**<br>**8.96**	**392**<br>**16.85**
Cardiovascular problems	**258**<br>**18.09**	**49**<br>**7.74**	**13**<br>**4.85**	**320**<br>**13.75**
Worsening of general condition	**61**<br>**4.28**	**15**<br>**2.37**	**12**<br>**4.48**	**88**<br>**3.78**
Neurological problem	**21**<br>**1.47**	**8**<br>**1.26**	**6**<br>**2.24**	**35**<br>**1.50**
Other	**133**<br>**9.33**	**44**<br>**6.95**	**12**<br>**4.48**	**189**<br>**8.12**
Post-operative visits	**23**<br>**1.61**	**15**<br>**2.37**	**5**<br>**1.87**	**43**<br>**1.85**
Bad laboratory results	**45**<br>**3.16**	**10**<br>**1.58**	**3**<br>**1.12**	**58**<br>**2.49**
Infections	**15**<br>**1.05**	**13**<br>**2.05**	**5**<br>**1.87**	**33**<br>**1.42**
	1,426<br>100	633<br>100	268<br>100	2,327<br>100

Table 1 List of condition which lead to the visit to emergency services per migration status
