# Supplementary material for: Adequate Utilization of Emergency Services in Germany: Is There a Differential by Migration Background?
Source: Front Public Health. 2021 Jan 8;8:613250. doi: 10.3389/fpubh.2020.613250 (PMC7820806; doi:10.3389/fpubh.2020.613250)
Supplement: Supplementary file 2 [file Data_Sheet_2.PDF]

**Adequate use of services**	**Model 1** (N=2 287)			**Model 1** (N=2 287)			**Model 2*** (N=2 287)			**Model 3*** (N=2 271)		
	OR	95% CI**	p-value	OR	95% CI**	p-value	OR	95% CI**	p-value	OR	95% CI**	p-value
Migration (reference: non-migrant)												
1st generation	0.72	[0.57, 0.91]	0.005	0.78	[0.62, 0.99]	0.046	0.78	[0.61, 0.99]	0.047	0.77	[0.60, 0.99]	0.046
2nd generation	0.76	[0.53, 1.09]	0.131	0.80	[0.56, 1.15]	0.231	0.76	[0.50, 1.05]	0.093	0.72	[0.50, 1.03]	0.077
Gender (Male)	1.28	[1.05, 1.56]	0.017	1.06	[1.02, 1.14]	0.615	1.03	[0.96, 1.44]	0.149	1.03	[0.83, 1.29]	0.762
Age	1.03	[1.03, 1.04]	<0.001	1.03	[1.02, 1.03]	<0.001	1.03	[1.03, 1.04]	<0.001	1.02	[1.01, 1.03]	<0.001
Nb. of chronic diseases (reference: none)												
1	1.03	[0.81, 1.31]	0.809	1.01	[0.79, 1.29]	0.925	0.99	[0.79, 1.30]	0.908	0.98	[0.76, 1.26]	0.859
2	1.25	[0.94, 1.68]	0.127	1.24	[0.92, 1.66]	0.151	1.20	[0.91, 1.66]	0.912	1.15	[0.87, 1.60]	0.358
3	1.53	[1.12, 2.09]	0.008	1.52	[1.11, 2.08]	0.009	1.47	[1.10, 2.11]	0.011	1.39	[1.00, 1.90]	0.052
Type of facility (gynaecology/reference : internal medicine)				0.53	[0.40, 0.69]	<0.001	0.48	[0.37, 0.68]	<0.001	0.48	[0.33, 0.69]	<0.001
Reasons for choice of facility (reference: sent by medical staff)												
Proximity of location							0.70	[0.55, 0.88]	0.003	0.70	[0.55, 0.89]	0.004
Reputation/recommendation							0.75	[0.45, 1.23]	0.253	0.79	[0.47, 1.30]	0.342
Known or relevant facility							0.85	[0.66, 1.09]	0.205	0.87	[0.67, 1.12]	0.272
Other							0.68	[0.29, 1.57]	0.365	0.70	[0.30, 1.65]	0.416
Evening/night							0.89	[0.63, 1.27]	0.537	0.92	[0.65, 1.32]	0.654
Education (reference: medium)												
Low										0.69	[0.53, 0.89]	0.005
High										0.77	[0.60, 0.96]	0.023
Employed (reference: unemployed)										0.68	[0.54, 0.86]	0.002

Supplementary Table: Results of the regression analysis. All variables are patient reported to the exception of type of facility and Evening/night, which was provided by the interviewer.* Models 2 to 4 is also adjusted for the type of condition that motivated the visit to the emergency services (13 categories). ** Confidence interval.
